# Medical student wellbeing – a consensus statement from Australia and New Zealand

**DOI:** 10.1186/s12909-019-1505-2

**Published:** 2019-03-04

**Authors:** Sandra Kemp, Wendy Hu, Jo Bishop, Kirsty Forrest, Judith N. Hudson, Ian Wilson, Andrew Teodorczuk, Gary D. Rogers, Chris Roberts, Andy Wearn

**Affiliations:** 1 Curtin Medical School, Faculty of Health Sciences, Curtin University, Perth, Western Australia; 2 School of Medicine, Western Sydney University, Penrith, New South Wales Australia; 3 Faculty of Health Sciences and Medicine, Bond University, Gold Coast, Queensland Australia; 4 Faculty of Health and Medical Sciences, University of Adelaide, Adelaide, South Australia; 5 School of Medicine, Faculty of Science, Medicine and Health, University of Wollongong, Wollongong, New South Wales Australia; 6 School of Medicine, Griffith University, Gold Coast, Queensland Australia; 7 Northern Clinical and Sydney Medical School, Faculty of Medicine and Health, The University of Sydney, Sydney, New South Wales Australia; 8 Medical Programme Directorate, Faculty of Medical & Health Sciences, University of Auckland, Auckland, New Zealand

**Keywords:** Medical student, Trainee, Well-being, Curriculum, Learning, Assessment, Psychological stress

## Abstract

**Background:**

*Medical student wellbeing* ***–*** *a consensus statement from Australia and New Zealand* outlines recommendations for optimising medical student wellbeing within medical schools in our region. Worldwide, medical schools have responsibilities to respond to concerns about student psychological, social and physical wellbeing, but guidance for medical schools is limited. To address this gap, this statement clarifies key concepts and issues related to wellbeing and provides recommendations for educational program design to promote both learning and student wellbeing. The recommendations focus on student selection; learning, teaching and assessment; learning environment; and staff development. Examples of educational initiatives from the evidence-base are provided, emphasising proactive and preventive approaches to student wellbeing.

**Main recommendations:**

The consensus statement provides specific recommendations for medical schools to consider at all stages of program design and implementation. These are:

- Design curricula that promote peer support and progressive levels of challenge to students.
- Employ strategies to promote positive outcomes from stress and to help others in need.
- Design assessment tasks to foster wellbeing as well as learning.
- Provide mental health promotion and suicide prevention initiatives.
- Provide physical health promotion initiatives.
- Ensure safe and health-promoting cultures for learning in on-campus and clinical settings.
- Train staff on student wellbeing and how to manage wellbeing concerns.

**Conclusion:**

A broad integrated approach to improving student wellbeing within medical school programs is recommended. Medical schools should work cooperatively with student and trainee groups, and partner with clinical services and other training bodies to foster safe practices and cultures. Initiatives should aim to assist students to develop adaptive responses to stressful situations so that graduates are prepared for the realities of the workplace. Multi-institutional, longitudinal collaborative research in Australia and New Zealand is needed to close critical gaps in the evidence needed by medical schools in our region.

## Background

Educators in medical schools worldwide are concerned about ensuring the psychological, social and physical wellbeing of medical students. In Australia, recent reports of tragic events related to doctors and mental health [1, 2] have triggered greater scrutiny of stress experienced by the medical profession and, in particular, doctors in training. Medical students are reported to have higher levels of alcoholism, relationship problems, depression and motor vehicle accidents [3]. Some studies highlight disquieting levels of burnout [4] and psychological distress in medical students [5]. Of particular concern for medical practice is the well documented negative impact of medical student distress and burnout on professional behaviour, ethical conduct and empathy [6].

Accreditation standards for Australia and New Zealand, United Kingdom (UK) and North American medical schools mandate the provision of student support [7–9], and best practice guidelines exist for supporting medical students with mental health concerns in the UK [10]. However, there has been no collective statement which sets out recommendations for practice in Australia and New Zealand. Medical schools have a responsibility to respond to concerns about medical student wellbeing because of the effects on individuals [11] and their capacity to learn. Other important drivers are the resource implications for government and medical schools for training not completed [12] and potential adverse consequences for patient care [13]. To address these concerns, this statement outlines recommendations for educational program design and student wellbeing that focus on: student selection; learning, teaching and assessment; learning environment; and staff development.

Clarity around the definition of wellbeing is elusive, with numerous terms and dimensions used, sometimes interchangeably. For this statement, *wellbeing* is defined as “when individuals have the psychological, social and physical resources they need to meet a particular psychological, social and/or physical challenge” ([14] p230). If individuals believe their own resources are sufficient, a stress response is not generated [15]. When a stress response or distress is generated due to beliefs that one’s own resources are insufficient to meet demands, there are detrimental effects on memory and reasoning [16], symptoms of anxiety or depression [4, 17] and burnout [4, 18]. Recent research also suggests that an individual’s mindset about stress plays a role in stress responses [19]. A stress mindset is a belief that stress has either enhancing or debilitating consequences for outcomes such as performance and learning. A mindset of stress-as-enhancing appears to be important for positive outcomes related to wellbeing [20].

Theoretical and empirical papers on medical student wellbeing have drawn attention to the construct of *resilience*—the ability to recover from setbacks when faced with adversity or stress [21]—which arguably, if nurtured, will improve wellbeing. Other studies have focussed on whether the personal characteristics of students and the ability to cope with stress have changed in recent years, or whether this is a values-based judgement from older generations about younger generations [22]. Evidence for either position is not yet conclusive, but such foci imply that problems, and thus the solutions, arise from individuals [23]. As medical educators, we advocate for a broader, more integrated approach to improving wellbeing, which actively considers changes at individual, institution and system levels. This statement focusses on individual and institution levels, where the evidence base in the medical education literature is primarily located.

Complex and uncertain environments where patients’ lives may be at stake, and where families, carers and many healthcare teams are involved, are inherently stressful but also provide unparalleled opportunities to learn. There are, however, also indefensible and avoidable stressors in medical practice and training (such as bullying by supervisors and excessive shift durations) which warrant urgent attention. Medical schools have a responsibility to work with other institutions to mitigate avoidable and unacceptable stressors, address the hidden curriculum and its impact on wellbeing, as well as to select and prepare students who will be equipped with appropriate knowledge, skills and professional behaviours to adaptively respond to the demands of the healthcare workplace.

As educators in medical schools, we present this consensus statement to continue dialogue about how medical programs can promote psychological, social and physical wellbeing. We primarily focus on psychological wellbeing; aspects of social and physical wellbeing, and references to other dimensions of wellbeing (such as spiritual) are included, but a comprehensive review of these are beyond the scope of this consensus statement. A statement on the relationship between fitness to practice and assessing professional behaviour in our region has been covered elsewhere [24]. Our statement focuses on wellbeing promotion and preventive approaches and the evidence-base. Our recommendations are not intended to substitute for, but to complement, university-wide student mental health support initiatives, such as the recent Enhancing Student Wellbeing project [25].

## Recommendations

Below we outline educational initiatives to promote wellbeing that should be considered by medical schools, and refer to evidence for these recommendations, where available.

### Student selection

Selection of students into medical school should reflect the demands of a career in medicine. It is possible to select for personal qualities which could assist in managing such challenges, but evidence for the reliable prediction of such outcomes is limited.

- Consider selection methods that have been shown to identify qualities appropriate for the challenges of study and training in medicine:
  While intuitively desirable, the evidence for selecting qualities such as tolerance of ambiguity [26] and the effect on subsequent performance is limited. Understanding of best practice in selection is still evolving [27–29]. There is uncertainty about the appropriate choice of instrument to measure desired attributes and whether such attributes are context specific or persistent traits that are demonstrable in the range of situations students will encounter.

### Learning, teaching and assessment

The formal program of learning, teaching and assessment should incorporate design principles that foster wellbeing, optimise responses to stress that motivate learning and provide appropriate support for each stage. Normalising discussions about self-care, wellbeing and personal impact on performance should underpin all aspects of student support programs. Creating opportunities for students to identify and appraise their own levels of stress, learn self-care strategies appropriate to their needs, and understand their professional responsibilities for promoting wellbeing are important.

b) Design curricula that promote peer support and develop healthy communities of practice:
  In the early years, small group collaborative activities such as problem-based learning and clinical skills teaching can provide continuity of peer group and establish tutors as role models [30]. In the clinical years, longer rotations, or longitudinal integrated clerkships [31] provide mechanisms for peer networks and continuity of clinical supervision to support learning and personal development. Such approaches can increase the sense of connectivity that underpins wellbeing [32].
c) Offer progressive levels of challenge to prepare for troubling encounters:
  Challenges within a supportive and collegial environment should be deliberately built into the curriculum in a graduated fashion, and can be supported by simulation activities such as learning how to break bad news [33]. Exposure to troubling encounters (such as the first cadaveric anatomy class) and transitions (such as from classroom to clinical learning) should be preceded by preparatory activities [34]. Experiences of communicating with distressed or angry patients, discussing sensitive issues and encounters with death can be aligned with activities to develop strategies for future encounters. Such activities include multimethod simulation [35], interprofessional education [36] and opportunities to debrief [37].
d) Offer strategies to build adaptive responses to stress:
  Examples that focus on positive outcomes from demanding situations include mentoring [38] and mindfulness training [39, 40]. Cognitive Behavioural Therapy [41] to reframe adversity may also be effective and is now freely available online (for example, https://moodgym.com.au). Initiatives should include increasing awareness of the impact of alcohol, prescribed and recreational drugs on learning and mood.
e) Implement personal physical health practices:
  Evidence that regular moderate-to-vigorous physical activity and nutrition reduce the risk of depression and feelings of anxiety [42–44] highlights an important link between physical health and psychological wellbeing. To complement the scientific study of exercise [45], nutrition [46] and medicine, personal physical health promotion activities for students can be added to motivate students to adopt healthier lifestyle habits [47, 48]. Examples include portfolio activities such as personal logs of diet and exercise [49] and the creation of exercise and nutrition plans. Optional health promoting extracurricular activities on-campus and in clinical settings such as exercise classes, healthy cooking classes, the use of personal fitness devices and apps, and modelling by staff, will reinforce medical school and medical profession support for healthy life [43, 50].
f) Encourage self-care and help giving:
  Mentoring and professional development curricula can provide opportunities for students to identify and appraise their own levels of stress, and to learn self-care strategies appropriate to their needs. Students should be urged to have their own general practitioner (GP) [51] as part of their professional responsibility to maintain their health. Lists of accessible, local GPs can be provided. Evidence shows that higher levels of psychological distress symptoms are associated with higher levels of reluctance to seek help [52]. Teaching students strategies for reaching out to peers in need of help with self-care can bridge this gap and build a community of support.
g) Bolster student support, independent from curriculum and assessment:
  Medical schools are required to provide safe mechanisms for students to seek personal advice [7] that will not impact on their progression. Options for student support include mentors who are adjunct staff or staff not involved in teaching or assessment, and providing information about external resources [53] such as independent medical and counselling services.
h) Design assessment tasks for optimal responses to stress and effective learning:
  Assessment tasks can be designed to foster capabilities for resilience, or the ability to recover from setbacks [54]. Reflection and feedback activities that focus on opportunities to learn, while building on strengths, can assist. Techniques can include relatively simple interventions such as timely debriefing after an OSCE, a stressful performance assessment. Substantive changes at the program level include forms of longitudinal assessment with a focus on the continuity of feedback [55, 56]. Portfolios [57] or grading that de-emphasises comparisons with others [58] also support learning.
i) Encourage exploration of meaning and purpose in studying medicine:
  The “search for meaning, purpose and connectedness” in life [59 p10], referred to by some as spirituality, can help some students positively reframe stressful situations [59], negative personal life events [60] or troubling patient care experiences [61]. For some students, spirituality can support coping strategies to overcome the challenges of studying medicine [62]. Reflective activities enable students to explore and understand the meaning and purpose of difficulties faced during educational experiences. Examples include a teaching OSCE to develop skills to reflect on personal sources of meaning during encounters with patients’ beliefs [63] or interprofessional education with theology students [64] or philosophers. Curriculum on the moral and spiritual issues related to caring for critically ill patients and the self-care they mandate have been explored [65].

### Learning environment

Creating safe environments for learning, where students understand that their concerns will be heard, is recommended. The student and trainee voice is critical to inform strategies to support ongoing learning and development. Anticipatory guidance about the junior doctor training experience should include wellbeing in the workplace.

j) Reduce barriers to seeking help:
  Stigma concerning mental health disorder or illness amongst medical students and the medical profession is common and a barrier to help seeking [66]. Studies in different contexts suggest that stigma may be more acute in some cultural groups and communities [67, 68]. Overcoming a culture of stigma by providing role models, addressing concerns about confidentiality and impact on progression and future career [69], and encouraging uptake of options such as online anonymous counselling will increase accessibility.
k) Engage students as stakeholders:
  Through regular contact with student representatives, medical schools can be alerted to emerging and collective concerns. This enables these concerns to be addressed pre-emptively. For example, concerns about mandatory reporting of impairment [1] can be a call to address misconceptions and provide positive information about seeking help. Schools can partner with student societies and the general student body to inform, educate and advocate for change. All Australian and New Zealand medical schools have national medical students’ association representatives, and often, a student wellbeing officer [70].
l) Implement and monitor policies to promote wellbeing:
  Policies related to suicide prevention should be implemented, and interventions that have not been shown to do harm should be established, in consultation with suicide prevention experts. Programs that promote mental health by fostering stress-as-enhancing mindsets appear important [19]. Programs teaching appropriate responses to the presence of suicidality can be implemented. Pro-actively communicating with students as a key audience when distressing events occur, such as the death of a peer or teacher, is widely practiced. Evidence suggests that participating in debriefing sessions should be an optional activity [71].
m) Promote safe cultures:
  Making it known that behaviours such as sexual harassment [72], bullying and discrimination, including that against LGBTI (Lesbian, Gay, Bisexual, Trans and Intersex) students, are not tolerated is critical. Suggested strategies from the Australian Human Rights Commission [73] should be adopted, including better communication of support available and taking whistleblowing by students seriously. Medical schools should have clear processes for how students can report incidents throughout their education and training and how such incidents will be handled, including consequences for inappropriate behaviours in themselves and others. Attention to behaviours that inadvertently reinforce human tendencies to focus on negative aspects, or “negativity bias” [74], and adoption of practices from positive psychology [75] may be helpful.

### Staff development

It is mandatory for medical schools to provide a system of student support [7]. However, both academic and professional staff often lack training in student support so staff development is necessary for the successful implementation of wellbeing and support programs.

n) Train staff on wellbeing:
  Professional and academic staff need training to be knowledgeable about contemporary approaches to student wellbeing [76] and to appreciate the impact on learning and performance. Educators with clinical backgrounds may have trained at a time when such concerns were less well documented, but may also have valuable experiences to draw upon as teachers and role models.
o) Train staff to manage wellbeing concerns:
  As well as training in curriculum and teaching that supports wellbeing, academic and professional staff are likely to need training in managing student presentations, difficult conversations and disclosures [77]. Staff also need to recognise boundaries between their professional role and personal motivations to help students, and when it is appropriate to refer students to other services.
p) Support staff wellbeing:
  Staff who interact with students with wellbeing concerns may be in need of support themselves. Access to, and encouragement to seek, staff counselling, peer support and other services should be provided [78]. Otherwise, informal messages that form part of the hidden curriculum and a culture of not attending to self-care may inadvertently be communicated, and should be addressed through staff development initiatives to assist staff with their own wellbeing [79].

## Conclusions

We have outlined recommendations for medical schools to consider when delivering programs that aim to optimise student wellbeing. A sound evidence base for these recommendations is yet to be developed so medical schools should take into account needs, acceptability and feasibility, as well as emerging evidence. There are significant gaps in evidence to inform student selection and curriculum/assessment design. Research to date has tended to be cross sectional, using quantitative measures of wellbeing related constructs (for example, [18]) or case studies of interventions in single institutions (for example, [80]) which may not be transferable to different medical schools or to postgraduate training programs.

In some regions, including Australia and New Zealand, multicentre, longitudinal studies are needed (similar to those in other countries [81]) across the continuum of medical education and training. Such studies will provide more direct evidence about the antecedents, promotors, and challenges to wellbeing and the effects of any interventions. We suggest that such collaborations could be led by bodies with sector oversight and responsibility for accredited medical education and training. In our region, this includes bodies such as the Medical Deans Australia and New Zealand, the peak body for all accredited medical schools, state health departments responsible for pre-specialty training, and specialty training Colleges.

In such collaborations, the student and trainee voice is critical. In 2013, the Australian Medical Students’ Association (AMSA) called for universities to maximise student wellbeing and provide preventative and intervention strategies. Junior doctors and trainees have initiated projects to document and bring attention to their experiences in the healthcare workplace (for example, [82]). Medical schools should partner with student and trainee representative councils and societies to design and conduct research and implement interventions that will help students to meet challenges to wellbeing as well as advocate for systems change [83].

In conclusion, medical schools and educators have a key role to play in developing and implementing strategies to support and prepare students for the inevitable stresses of real world practice rather than simply focussing on minimising stress. Educators should guard against graduating students who are yet to develop the capacity to cope with stress and challenge. Through the use of evidence-based educational strategies, educators can work with individuals, institutions and systems to promote wellbeing, learning and growth, for students, the profession and, ultimately, the patients they serve.

### Developing the consensus statement

Medical education leaders, representing 17 Australia and New Zealand medical schools, participated in the Medical Education Collaboration Committee (MECC) – Medical Education Leads in Australian and New Zealand (MELANZ) Symposium, Adelaide, South Australia, on 10 July 2017. During this event, facilitated roundtable discussions were conducted on the role of medical education in student wellbeing, generating written summaries of agreed issues and responses. Qualitative data from these discussions were analysed thematically by the authors to generate preliminary concepts for this statement. Recommendations were then developed with reference to evidence from peer-reviewed studies in medical education, within the limitation that these were not based on systematic review protocols or stakeholder viewpoints other than medical educators and deans of medical schools. The consensus statement was finalised following three cycles of review by the authors and other lead medical educators as expert reviewers, review by the Executive and all members of Medical Deans Australia and New Zealand, and endorsed by Medical Deans.

## Abbreviations

AMSA: Australian Medical Students’ Association
GP: General Practitioner
LGBTI: Lesbian, Gay, Bisexual, Trans and Intersex
MECC: Medical Education Collaboration Committee
MELANZ: Medical Education Leads in Australian and New Zealand
OSCE: Objective Structured Clinical Examination
UK: United Kingdom
